# Coherent spin dynamics of electrons and holes in CsPbBr_3_ perovskite crystals

**DOI:** 10.1038/s41467-019-08625-z

**Published:** 2019-02-08

**Authors:** Vasilii V. Belykh, Dmitri R. Yakovlev, Mikhail M. Glazov, Philipp S. Grigoryev, Mujtaba Hussain, Janina Rautert, Dmitry N. Dirin, Maksym V. Kovalenko, Manfred Bayer

**Affiliations:** 1 Experimentelle Physik 2, Technische Universität Dortmund, D-44221 Dortmund, Germany; 2 Ioffe Institute, Russian Academy of Sciences, 194021 St. Petersburg, Russia; 3 Spin Optics Laboratory, St. Petersburg State University, 199034 St. Petersburg, Russia; 4 Centre for Micro and Nano Devices, Department of Physics, COMSATS University, 44000 Islamabad, Pakistan; 5 Laboratory of Inorganic Chemistry, Department of Chemistry and Applied Biosciences, ETH Zürich, CH-8093 Zürich, Switzerland; 6 Laboratory for Thin Films and Photovoltaics, Empa-Swiss Federal Laboratories for Materials Science and Technology, CH-8600 Dübendorf, Switzerland

## Abstract

The lead halide perovskites demonstrate huge potential for optoelectronic applications, high energy radiation detectors, light emitting devices and solar energy harvesting. Those materials exhibit strong spin-orbit coupling enabling efficient optical orientation of carrier spins in perovskite-based devices with performance controlled by a magnetic field. Here we show that elaborated time-resolved spectroscopy involving strong magnetic fields can be successfully used for perovskites. We perform a comprehensive study of high-quality lead halide perovskite CsPbBr_3_ crystals by measuring the exciton and charge carrier *g*-factors, spin relaxation times and hyperfine interaction of carrier and nuclear spins by means of coherent spin dynamics. Owing to their ‘inverted’ band structure, perovskites represent appealing model systems for semiconductor spintronics exploiting the valence band hole spins, while in conventional semiconductors the conduction band electrons are considered for spin functionality.

## Abstract (web summary)

Despite remarkable optical properties in lead halide perovskites, spin control in these materials is largely unexplored. Herein Belykh et al. study the coherent spin dynamics of electrons and holes in cesium lead bromide perovskites, and evidence interaction of electron and lattice nuclear spins.

## Introduction

Semiconductor spintronics is an intense research field covering the whole variety of spin-dependent phenomena and numerous experimental techniques, which allow one to study the spin structure and spin dynamics in different materials and their nanostructures. Optical techniques with time- and polarization resolution and application of magnetic field are widely used for that. Despite the great recent interest to various perovskite materials^1–4^, including two-dimensional perovskites and colloidal nanocrystals, spin studies are at the very beginning here, while substantial bulk and structure inversion asymmetry^5–7^ make perovskites promising for spintronics^8,9^. It has been demonstrated, however, that experimental approaches like optical orientation^10^, spin polarization induced by magnetic field^11,12^, pump–probe Faraday rotation^13,14^, and single dot spectroscopy in magnetic field^15–17^ are working well for perovskites and their nanostructures. The fine structure of neutral and charged excitons has been addressed, including their spin dynamics. Recently, it has been shown that the combination of spin–orbit and exchange interactions in perovskite nanocrystals may result in an unusual ordering of the exciton fine structure levels with an optically active ground state^18^.

Here, we report spin-dependent phenomena in CsPbBr_3_ perovskite crystals of high structural and optical quality, as confirmed by sharp exciton resonances in reflectivity and emission spectra. We focus on the coherent spin dynamics in external magnetic fields at cryogenic temperatures studied by optical techniques based on the pump–probe time-resolved Kerr rotation. We measure the transverse and longitudinal spin relaxation times of electrons and holes and their dependencies on magnetic field and temperature. We evaluate the exciton, electron and hole *g*-factors including their signs and spread. Polarizing the nuclear spins dynamically via optically oriented carriers, we address hyperfine interaction effects and find the dominant role of the holes in them, which is in agreement with our model considerations.

## Results

### Optical characterization

**Fig. 1:**
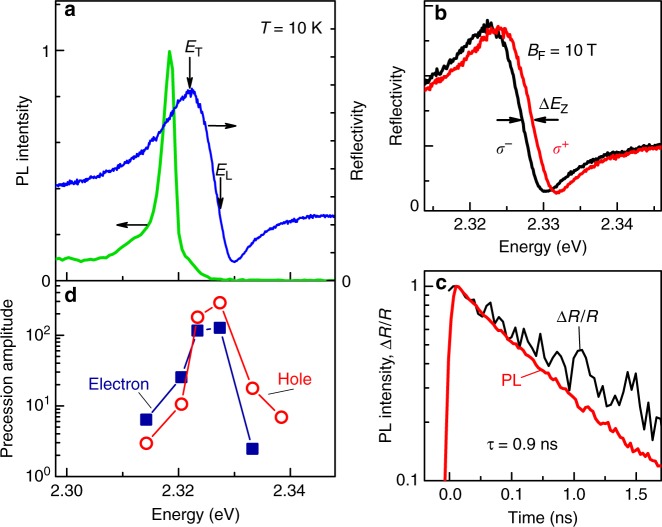
Photoluminescence and reflectivity of CsPbBr_3_ perovskite crystal. **a** Photoluminescence (green line, excitation energy at 2.376 eV) and reflectivity (blue line) spectra. Energies for longitudinal (*E*_L_) and transverse (*E*_T_) exciton-polaritons are marked by arrows. **b** Reflectivity spectra measured for opposite circular polarizations in longitudinal magnetic field *B*_F_ = 10 T. **c** Exciton recombination dynamics measured at 2.318 eV with streak-camera under nonresonant excitation (at 3.263 eV, red line) and measured under resonant excitation at 2.328 eV as the signal of differential reflection (black line). *T* = 10 K. **d** Spectral dependence of spin precession amplitude of electrons (solid squares) and holes (open circles) in transverse magnetic field *B*_V_ = 0.5 T. Source data are provided as a Source Data file

We start with optical characterization of the CsPbBr_3_ perovskite crystal at a low temperature of 10 K. The reflectivity spectrum shown in Fig. 1a by the blue line demonstrates a strong exciton–polariton resonance with transverse and longitudinal energies of *E*_T_ = 2.3220 eV and *E*_L_ = 2.3274 eV (Methods). In a longitudinal magnetic field of *B*_F_ = 10 T the reflectivity spectra measured in the two opposite circular polarizations show an exciton Zeeman splitting of Δ*E*_Z_ = 1.32 meV (Fig. 1b), which corresponds to the exciton *g*-factor *g*_X_ = Δ*E*_Z_/(*μ*_B_*B*_F_) = 2.35, where *μ*_B_ is the Bohr magneton. In the photoluminescence (PL) spectrum (the green line in Fig. 1a) the narrow exciton peak at 2.318 eV has a small Stokes shift of 4 meV from *E*_T_. The PL and reflectivity spectra are in agreement with previous studies for this material^19^. The exciton has a lifetime of 0.9 ns measured by differential reflection Δ*R*/*R* dynamics, which is very close to the decay time of time-resolved PL of 0.7 ns (Fig. 1c). The PL band at the lower energy side of the exciton line presumably arises due to bound excitons, its PL dynamics is presented in the Supplementary Note 6 and is not much longer than the exciton PL dynamics.

### Coherent spin dynamics of electrons and holes

**Fig. 2:**
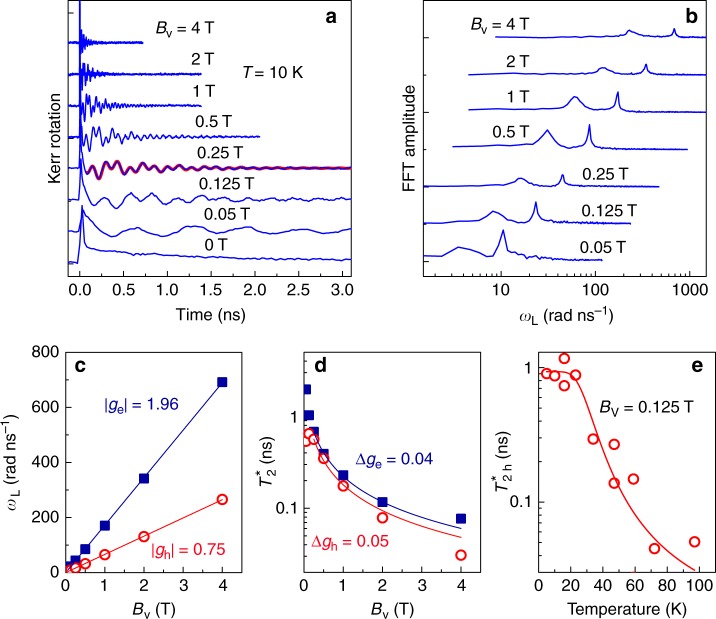
Coherent spin dynamics in transverse magnetic field. **a** Kerr rotation dynamics in CsPbBr_3_ crystal at different magnetic fields. Magenta thick line for *B*_V_ = 0.25 T is fit to the experimental data with two decaying oscillatory functions (Methods). **b** Fast Fourier transform spectra of spin dynamics traces from **a**. **c** Magnetic field dependencies of electron (squares) and hole (circles) Larmor frequencies. Lines show linear fits to data. **d** Magnetic field dependencies of electron (squares) and hole (circles) spin dephasing times. Lines show reciprocal to *B*_V_ fits to data with evaluated spread of *g*-factors. **a**–**d** *T* = 10 K. **e** Temperature dependence of hole spin dephasing time. Line is fit with activation dependence having energy parameter Δ*E* = 14 meV. Source data are provided as a Source Data file

The coherent spin dynamics of carriers is measured by the time-resolved pump–probe Kerr rotation. Figure 2a shows the spin dynamics at different magnetic fields *B*_V_ applied perpendicular to the pump and probe beams (Voigt geometry). The oscillating signals result from the Larmor precession of the carrier spin polarization about the magnetic field with frequency *ω*_L,e(h)_ = |*g*_e(h)_|*μ*_B_*B*_V_/*ħ*^20,21^. Here, *g*_e_ and *g*_h_ are the electron and hole *g*-factors, respectively. The signal precession is seemingly aperiodic, which is due to the presence of two frequencies, as evidenced from two peaks in the fast Fourier transform (FFT) spectra of the spin dynamics (Fig. 2b). The two frequencies increase linearly with magnetic field (Fig. 2c) and correspond to *g*-factors of |*g*_e_| = 1.96 and |*g*_h_| = 0.75. This assignment of the *g*-factors to electrons and holes is based on the following arguments. First, the exciton contribution is excluded as it should be characterized by a *g*-factor of *g*_X_ = 2.35 extracted directly from the Zeeman splitting in reflectivity. Second, both *ω*_L_(*B*_V_) dependencies can be extrapolated to zero frequency for vanishing field, i.e., no contribution of an exciton exchange splitting is seen^17^. Also, the spin dephasing times are longer than the 0.9 ns exciton lifetime. In the perovskites *g*_X_ = *g*_e_ + *g*_h_ (Supplementary Note 4) and we found experimentally that *g*_X_ > 0 and |*g*_e_|, |*g*_h_| < *g*_X_. Therefore, we conclude that in the studied material *g*_e_ > 0 and *g*_h_ > 0. A specifics of the perovskite band structure, compared to common II–VI and III–V semiconductors, is the strong renormalization of the hole *g*-factor compared to the electron one^8^. This allows us to assign the 1.96 *g*-factor to the electron, and the smaller *g*-factor to the hole. Note, that the *g*-factor depends on the band parameters, in particular, on the band gap. Due to substantial variation between the band gaps of various perovskite materials, the magnitude and sign of *g*-factor differ for them.

The electron and hole spin signals are maximal for laser energies close to the exciton–polariton resonance (Fig. 1d) due to efficient spin initialization and detection in resonance. The dephasing time of the spin precession, $T_2^ \ast$, shortens with increasing *B*_V_ (Fig. 2d), which is related to the spread of *g*-factor values, Δ*g*. It can be described by $1{\mathrm{/}}T_2^ \ast = 1{\mathrm{/}}\tau _{\mathrm{s}} + {\mathrm{\Delta }}g\mu _{\mathrm{B}}B_{\mathrm{V}}{\mathrm{/}}\hbar$, where *τ*_s_ is the spin lifetime at zero field. A fit with this equation gives Δ*g*_e_ = 0.04, *τ*_s,e_ = 2 ns, Δ*g*_h_ = 0.05, and *τ*_s,h_ = 1 ns.

We suggest that the spin dynamics is contributed by resident electrons and holes localized in CsPbBr_3_ at spatially separated locations. The resident carriers can be provided by unintentional doping in solution-grown crystals^22^ or by photogeneration, which is in line with the remarkable photovoltaic properties of perovskites. The lifetime of these carriers is significantly longer than the laser repetition period of 13.1 ns^1,23^. Therefore, the spin polarization can be accumulated from many subsequent pump pulses^21^. The mechanism of spin coherence generation for resident carriers is the same as in semiconductor quantum wells and quantum dots, see Supplementary Note 2 and refs. ^20,21^. As we show below, the established experimental approaches for spin dynamics studies in conventional semiconductor nanostructures are also suitable for the perovskites.

**Fig. 3:**
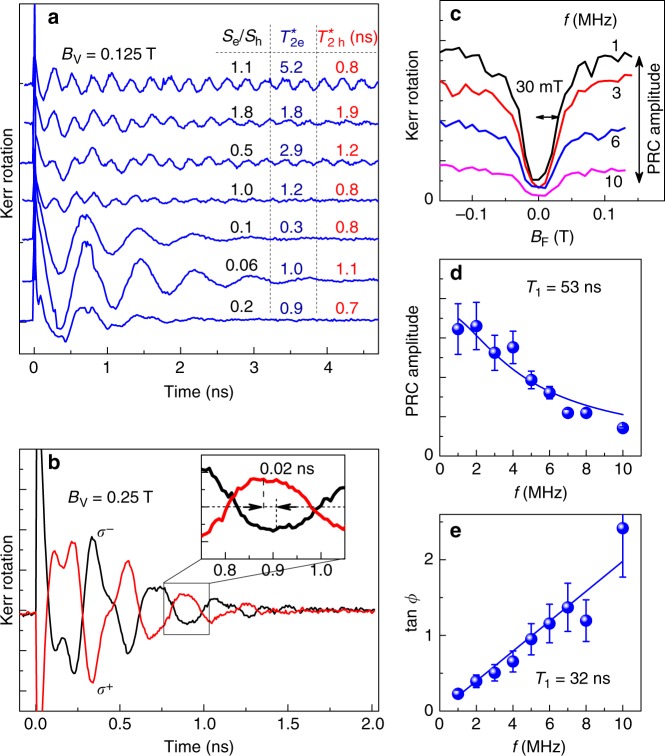
Carrier-nuclei hyperfine interaction and evaluation of longitudinal spin relaxation time *T*_1_. **a** Spin dynamics at different positions on sample, *B*_V_ = 0.125 T and *T* = 10 K. **b** Dynamics of Kerr rotation for different circular polarizations of pump pulses. Inset illustrates phase shift acquired for hole spin precession. *T* = 5 K. Pump is tilted from normal incidence by an angle of 15°. **c** Polarization recovery curves (PRCs): dependencies of Kerr rotation signal on longitudinal magnetic field at time delay Δ*t* = 13 ns, measured for different pump modulation frequencies. *T* = 2 K. **d** Modulation frequency dependence of PRC amplitude. Line is fit to data with Equation (2) giving *T*_1_ = 53 ns. **e** Modulation frequency dependence of tan *ϕ*, where *ϕ* is phase retardation of Kerr rotation signal with respect to pump modulation. Line is linear fit tan *ϕ* = 2*πfT*_1_ with *T*_1_ = 32 ns. Error bars represent the standard deviations. Source data are provided as a Source Data file

In the studied CsPbBr_3_ crystal the relative amplitudes of the electron and hole signals vary with laser spot position (Fig. 3a). This evidences the inhomogeneous spatial distribution of the resident carriers and confirms their localization. Note that the hole spin dephasing time at weak magnetic fields $( {T_{2,{\mathrm{h}}}^ {\ast} \approx \tau _{{\mathrm{s}},{\mathrm{h}}} \approx 1\,{\mathrm{ns}}} )$ is not very sensitive to the spot position. By contrast, the electron dephasing time $T_{2,{\mathrm{e}}}^ \ast$ has a stronger dependence on the spot position reaching up to 5.2 ns, which exceeds by almost an order of magnitude the lifetime of excitons (0.9 ns) and bound excitons (0.9 ns, see Supplementary Note 6). The dependence of $T_{2,{\mathrm{e}}}^ \ast$ on the spot position can be related to dependence of the strength of hyperfine interaction (see Supplementary Note 3) on the localization length. Moreover, the spin-flip mechanisms for localized charge carriers involving an interplay of the spin–orbit interaction and electron–phonon coupling are also strongly dependent on the parameters of localization^24^.

The electron and hole spin dephasing times are almost constant at temperatures less than 15 K. At higher temperatures the electron spin precession is not detectable due to the abrupt shortening of $T_{2,{\mathrm{e}}}^ \ast$. Hole spin dynamics can be measured up to 100 K and $T_{2,{\mathrm{h}}}^ \ast$ smoothly decreases with increasing temperature (Fig. 2e). This behavior can be described by an activation-type dependence: $1{\mathrm{/}}T_2^ \ast (T) = 1{\mathrm{/}}T_2^ \ast (0) + w\,{\mathrm{exp}}( - {\mathrm{\Delta }}E{\mathrm{/}}k_{\mathrm{B}}T)$. Here, $T_2^ \ast (0)$ is the spin dephasing time at zero temperature, *w* is a phenomenological prefactor, and *k*_B_ is the Boltzmann constant. The activation energy Δ*E* = 14 meV and *w* = 160 ns^−^^1^ are evaluated from the fit. This behavior can be related to either hole delocalization or to a spin-flip process mediated by LO phonons, whose energy in CsPbBr_3_ is about 18 meV^19^.

### Interaction of charge carriers with nuclear spins

In semiconductors the spin dynamics of localized carriers is mainly controlled by the carrier hyperfine interaction with the nuclear spins^25^, as the quenching of the orbital motion suppresses the spin–orbit coupling effects^24,25^. The nuclear spins experience the Knight field from the spin-polarized carriers and, in turn, the carriers experience the Overhauser field induced by the polarized nuclei. To examine nuclear effects, we intentionally polarize the nuclear spins by setting the circular polarization of the pump beam and tilting it by an angle of 15° from the normal to the sample surface. This provides a nonzero projection of the charge carrier spin polarization onto the magnetic field. Flip-flop hyperfine processes transfer the carrier spin polarization to the nuclear spin system. This gains a dynamical nuclear polarization *I* (Supplementary Note 3) and induces an Overhauser field *B*_N,e(h)_ = *A*_e(h)_*I*/(*g*_e(h)_*μ*_B_), that adds up to the external field. It changes the frequency of the carrier spin precession. The direction of the Overhauser field is determined by the pump helicity.

Figure 3b shows that the spin beats for opposite pump polarizations acquire a small but detectable relative phase shift, which increases with delay time, evidencing a difference in the spin precession frequencies. An accurate fit of the experimental data shows that the Overhauser field acting on the holes |*B*_N,h_| = 3.1 ± 0.5 mT is three times larger than that on the electrons |*B*_N,e_| = 1.0 ± 0.8 mT. This result may seem surprising compared with the widely studied III–V and II–VI semiconductors where the hyperfine coupling is dominated by the conduction band electrons^26,27^. Our theoretical analysis demonstrates that the stronger hyperfine coupling for the valence band holes compared to the conduction band electrons is a particular feature of perovskites such as CsPbBr_3_ (Supplementary Note 3). We estimate that the hole hyperfine coupling by the Fermi contact interaction with the ^207^Pb isotopes with *I* = 1/2 amounts to *A*_h_ = 20 μeV. The somewhat weaker dipole–dipole interaction of the conduction band electron with the ^79^Br and ^81^Br isotopes with *I* = 3/2 gives *A*_e_ = 7 μeV. These estimates demonstrate that the dynamical nuclear polarization is far from 100%, most probably due to spin relaxation processes unrelated to the hyperfine coupling, see Supplementary Note 3 for details.

At low temperatures and weak magnetic fields the spin dephasing time $T_2^ \ast$ of localized charge carriers is mainly contributed by the static fluctuations of the nuclear Overhauser field. Application of a longitudinal magnetic field *B*_F_ (Faraday geometry) parallel to the initial spin polarization suppresses the transverse fluctuations and stabilizes carrier spins against the influence of random nuclear fields. Insight into the effect can be obtained from polarization recovery curves (PRC)^28^ (Fig. 3c). The polarization recovery for alternating optical orientation allows us also to evaluate the longitudinal spin relaxation time *T*_1_ of the charge carriers via the spin inertia method^28^ (Methods). Typical PRC show an increase of the spin polarization with increasing magnetic field exhibiting a half width at half maximum of 30 mT and saturation with growing *B*_F_ (Fig. 3c). The saturation level decreases with increasing the pump modulation frequency *f* from 1 to 10 MHz. Fitting of the dependence of the PRC amplitude on *f* with Eq. (2) (Methods) gives *T*_1_ = 53 ± 9 ns (Fig. 3d). The measurement of the frequency dependence of the phase retardation, *ϕ*, of the spin polarization signal with respect to that of the pump modulation allows us to evaluate *T*_1_ = 32 ± 2 ns using tan *ϕ* = 2*πfT*_1_ (Fig. 3e). The different *T*_1_ values evidence a nonmonoexponential decay of the spin polarization (Methods). We also find that *T*_1_ is constant for a temperature increase from 2 to 10 K, and then strongly decreases to a few ns for temperatures exceeding 20 K, presumably due to the same activation process as relevant for $T_{{\mathrm{2,h}}}^ \ast$ (Fig. 2e). The analysis of the PRC and spin inertia signals provides an estimate of the nuclear field fluctuations *δB*_N_ = 6.6 mT and the hole correlation time at the localization site *τ*_c_ = 2.1 ns (Supplementary Note 5).

## Discussion

In summary, we have demonstrated that spin phenomena show up as prominent features in the optical properties of perovskites, even though they have remained largely unexplored so far. Fortunately, the methodology established for other semiconductors can be transferred to the perovskites. In particular we have elaborated the importance of the nuclear spins in these phenomena, which may be used as additional resource, for example, for establishing a long-lived spin memory. Based on our results, one may seek also for spin–orbit effects in spin relaxation and decoherence and for coherent spin control in perovskites.

## Methods

### Growth of CsPbBr_3_ samples

Single crystals of CsPbBr_3_ were grown as reported elsewhere with slight modifications^22^. First, CsBr and PbBr_2_ were dissolved in dimethyl sulfoxide at concentrations of 0.5 and 1 M, respectively, and the resulting solution (2 mL) was filtered through polytetrafluoroethylene filter (0.2 μm). Totally, 2 mL of the cyclohexanol solution in N,N-dimethylformamide (5.1 g in 9.1 g, respectively) were added and the resulting mixture was heated in an oil bath to 70 °C and then slowly (about 0.05–0.1 °C min^−1^) to 105 °C. After about 12 h of growth, the obtained crystals were taken out of the solution and quickly loaded into a vessel with hot (100 °C) *N*,*N*-dimethylformamide. This vessel was slowly (about 25 °C h^−1^) cooled down to about 50 °C. After that the crystals were isolated, wiped with filter paper and dried. The obtained rectangular CsPbBr_3_ is crystallized in the orthorhombic modification. The crystals have a one selected (long) direction along the *c*-axis [001] and two nearly identical directions along the [${\bar{1}}10$] and [110] axes.

### Reflectivity and photoluminescence characterization

For optical experiments the CsPbBr_3_ sample was placed in a liquid-helium-cooled cryostat, where the sample temperature, *T*, was varied from 2 up to 100 K. The cryostat was equipped with a superconducting split-coil solenoid generating external magnetic fields up to 10 T, which were applied either parallel to the light wave vector in the Faraday geometry (*B*_F_), or perpendicular to it in the Voigt geometry (*B*_V_). The optical signals were dispersed with an 0.5-m spectrometer and detected by a liquid-nitrogen-cooled charge coupled device detector.

Reflectivity spectra were measured using a halogen lamp in back-reflected geometry. The exciton–polariton resonance was modeled by the approach of ref. ^29^ (details will be published elsewhere), from which the following parameters were evaluated: transverse exciton energy *E*_T_ = 2.3220 eV, longitudinal exciton energy *E*_L_ = 2.3274 eV, longitudinal-transverse splitting *ħω*_LT_ = 5.4 meV, and exciton damping *ħΓ* = 6.7 meV. The exciton *g*-factor of *g*_X_ = 2.35 was measured from the Zeeman splitting of oppositely circularly polarized reflectivity spectra in magnetic fields up to 10 T.

The photoluminescence (PL) was excited by a continuous-wave laser with a photon energy of 2.376 eV. Low-excitation densities not exceeding 10 W/cm^2^ were used.

### Time-resolved PL

The exciton recombination dynamics was measured from time-resolved PL excited with 1 ps laser pulses at 3.263 eV photon energy and detected with a streak-camera attached to an 0.5-m spectrometer. The overall time resolution was 20 ps.

### Pump–probe time-resolved Kerr rotation

A polarization-sensitive pump–probe Kerr rotation technique^21^ was employed to study the spin dynamics of carriers, for which magnetic fields up to *B*_V_ = 4 T were applied in the Voigt geometry, i.e., perpendicular to the sample normal and to the light propagation direction. The used laser system was composed of a pulsed Ti:Sapphire laser which pumps an optical parametric oscillator with intracavity second harmonics generation providing wavelength-tunable emission in the range of 500–800 nm with a spectral width of about 1 nm and a pulse duration of 1 ps. The pulse repetition rate was 76 MHz (repetition period *T*_R_ = 13.1 ns).

The output of the laser system was split into the pump and probe beams. The circularly polarized pump pulses create spin polarization of the carriers in the sample. The spin polarization was then analyzed by measuring the Kerr rotation of the linearly polarized probe pulses reflected from the sample. Varying the time delay between the pump and probe pulses by means of a mechanical delay line gave access to the time dependence of the spin polarization. The polarization of the pump beam was modulated between *σ*^+^ and *σ*^−^ by a photo-elastic modulator operated at a frequency of 84 kHz for synchronous detection. In finite magnetic field, the Kerr rotation amplitude oscillates in time reflecting the Larmor spin precession of the carriers and decays at longer time delays. When both electrons and holes contribute to the Kerr rotation signal, as is the case for the studied CsPbBr_3_ sample, the signal can be described with a superposition of two decaying oscillatory functions: *A*_KR_ = $S_{\mathrm{e}}\,{\mathrm{cos}}(\omega _{\mathrm{e}}t){\mathrm{exp}}( - t{\mathrm{/}}T_{2,{\mathrm{e}}}^ \ast )$ + $S_{\mathrm{h}}{\mathrm{cos}}(\omega _{\mathrm{h}}t){\mathrm{exp}}( - t{\mathrm{/}}T_{2,{\mathrm{h}}}^ \ast )$.

### Pump–probe time-resolved differential reflection

The exciton recombination dynamics under resonant exciton excitation at 2.328 eV was obtained by measuring the dynamics of differential reflection Δ*R*/*R* of the probe beam after excitation with the pump beam. This technique is similar to the above-described pump–probe Kerr rotation with the distinction that we registered changes in probe intensity, while the pump was linearly polarized (not orienting spins) and its intensity was modulated for synchronous detection.

### Polarization recovery measurements

Here, the pump–probe Kerr rotation was measured as well (see above), but the magnetic field *B*_F_ was applied in the Faraday geometry, i.e., parallel to the sample normal and light propagation direction. In order to detect spin dynamics of the resident carriers only and to avoid the contribution of excitons, the signal was detected at a time delay of 13 ns, i.e., shortly before the arrival of the next pump laser pulse^28^. The photogenerated carrier spin polarization is stabilized by the longitudinal magnetic field, which results in an increase of the Kerr rotation amplitude with growing magnetic field. Typical PRC saturates with increasing magnetic field (Fig. 3c). The difference between the saturated Kerr rotation signal and its value at zero field is the PRC amplitude. The width of the PRC provides information on the spin relaxation mechanisms and/or on the local magnetic fields, e.g., resulting from the nuclear spin fluctuations.

### Spin inertia method

The longitudinal spin relaxation time of the carriers, *T*_1_, was measured by the spin inertia method, which is based on the pump–probe Kerr rotation technique^28^. For that, the intensity of the circularly polarized pump was modulated with frequency *f*. When the modulation period 1/*f* exceeds *T*_1_, the carrier spin polarization follows the change of pump polarization. As the modulation frequency is increased, so that 1/*f* becomes comparable to *T*_1_, the spin polarization cannot follow the pump polarization and the Kerr rotation modulation amplitude decreases. Additionally, a phase retardation, *ϕ*, appears between the oscillations of the pump and carrier spin polarizations. One can show that for pumping with modulated polarization in the form *R*(*t*) = *R*_0_[1 + cos(2*πft*)], the carrier polarization takes the form

1 $$S(t) = R_0T_1 + S_{{\mathrm{ac}}}\,{\mathrm{cos}}(2\pi ft - \phi ),$$

where

2 $$S_{{\mathrm{ac}}} = \frac{{R_0T_1}}{{\sqrt {1 + \left( {2\pi T_1f} \right)^2} }},$$

and

3 $${\mathrm{tan}}\,\phi = 2\pi T_1f.$$

Equation (2) was derived in refs. ^28,30^, while Equation (3) was worked out in ref. ^31^. One can see, that *T*_1_ can be evaluated independently from the experimental dependences of *S*_ac_(*f*) and of *ϕ*(*f*). In case of a nonexponential spin dynamics the *T*_1_ values determined from Equations (2) and (3) will differ from each other, as the amplitude is more sensitive to the slower component, while the phase is dominated by the faster one. Note that by using the synchronous detection technique, in our experiments we detect only *S*_ac_, while the time-independent component of the spin polarization [first term in the right hand side of Equation (1)] is eliminated.

## Supplementary Information

Supplementary Information

Source Data

## Data Availability

The data that support the plots within this paper and other findings of this study are available from the corresponding authors upon reasonable request. The source data underlying Figs. 1–3 and Supplementary Figs. 1 and 2 are provided as a Source Data file.
