# Author Correction: Coherent spin dynamics of electrons and holes in CsPbBr3 perovskite crystals

**DOI:** 10.1038/s41467-019-09821-7

**Published:** 2019-04-15

**Authors:** Vasilii V. Belykh, Dmitri R. Yakovlev, Mikhail M. Glazov, Philipp S. Grigoryev, Mujtaba Hussain, Janina Rautert, Dmitry N. Dirin, Maksym V. Kovalenko, Manfred Bayer

**Affiliations:** 1 Experimentelle Physik 2, Technische Universität Dortmund, D-44221 Dortmund, Germany; 2 Ioffe Institute, Russian Academy of Sciences, 194021 St. Petersburg, Russia; 3 Spin Optics Laboratory, St. Petersburg State University, 199034 St. Petersburg, Russia; 4 Centre for Micro and Nano Devices, Department of Physics, COMSATS University, 44000 Islamabad, Pakistan; 5 Laboratory of Inorganic Chemistry, Department of Chemistry and Applied Biosciences, ETH Zürich, CH-8093 Zürich, Switzerland; 6 Laboratory for Thin Films and Photovoltaics, Empa-Swiss Federal Laboratories for Materials Science and Technology, CH-8600 Dübendorf, Switzerland

Correction to: *Nature Communications* 10.1038/s41467-019-08625-z, published online 08 February 2019

The original version of this Article contained an error in Fig. 2c, in which the numbers on the *y*-axis were given in the wrong order: ‘800’ at the bottom through to ‘0’ at the top. This has been corrected in both the PDF and HTML versions of the Article.

Also, the Source Data file initially published online was corrupted and was replaced.

