# Supplementary material for: Coherent spin dynamics of electrons and holes in CsPbBr3 perovskite crystals
Source: Nat Commun. 2019 Feb 8;10:673. doi: 10.1038/s41467-019-08625-z (PMC6368575; doi:10.1038/s41467-019-08625-z)
Supplement: Supplementary file 1 — Supplementary Information [file 41467_2019_8625_MOESM1_ESM.pdf]

Supplementary information: Coherent spin dynamics of electrons and holes in $CsPbBr_3$ perovskite crystals

Vasilii V. Belykh,[1] Dmitri R. Yakovlev,[1,2] Mikhail M. Glazov,[2] Philipp S. Grigoryev,[3] Mujtaba Hussain,[4] Janina Rautert,[1] Dmitry N. Dirin,[5] Maksym V. Kovalenko,[5,6] and Manfred Bayer[1,2]

[1]*Experimentelle Physik 2, Technische Universität Dortmund, D-44221 Dortmund, Germany*

[2]*Ioffe Institute, Russian Academy of Sciences, 194021 St. Petersburg, Russia*

[3]*Spin Optics Laboratory, St. Petersburg State University, 199034 St. Petersburg, Russia*

[4]*Centre for Micro and Nano Devices, Department of Physics, COMSATS University, 44000 Islamabad, Pakistan*

[5]*Laboratory of Inorganic Chemistry, Department of Chemistry and Applied Biosciences, ETH Zürich, CH-8093 Zürich, Switzerland*

[6]*Laboratory for Thin Films and Photovoltaics, Empa-Swiss Federal Laboratories for Materials Science and Technology, CH-8600 Dübendorf, Switzerland*

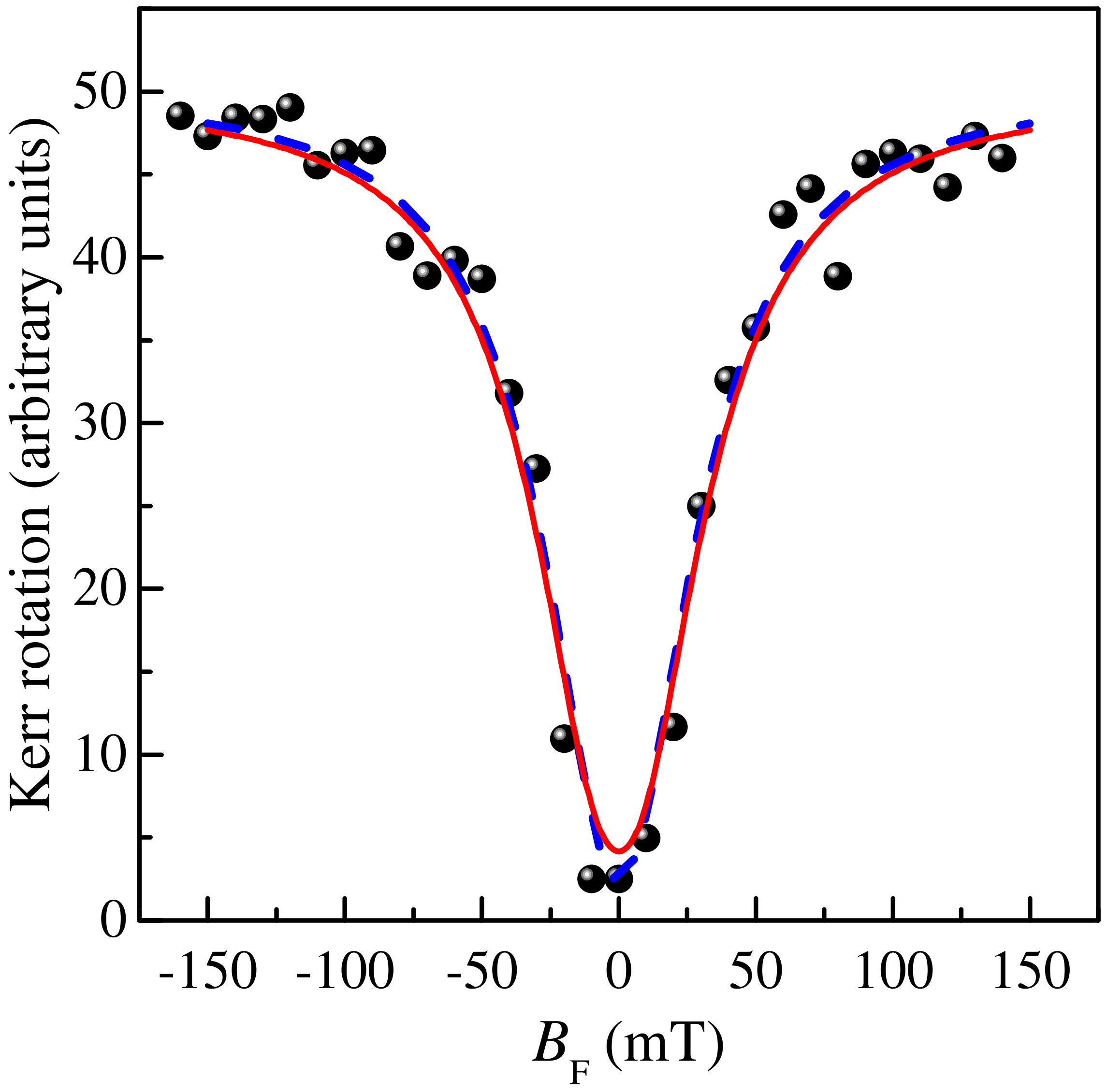

Supplementary Figure 1: Polarization recovery curve (PRC) measured at lowest modulation rate of 1 MHz together with fit after simplified Eq. (14) (blue dashed line) and results of calculation after the full model of Ref. 20 (red line), see text for parameters. Source data are provided as a Source Data file.

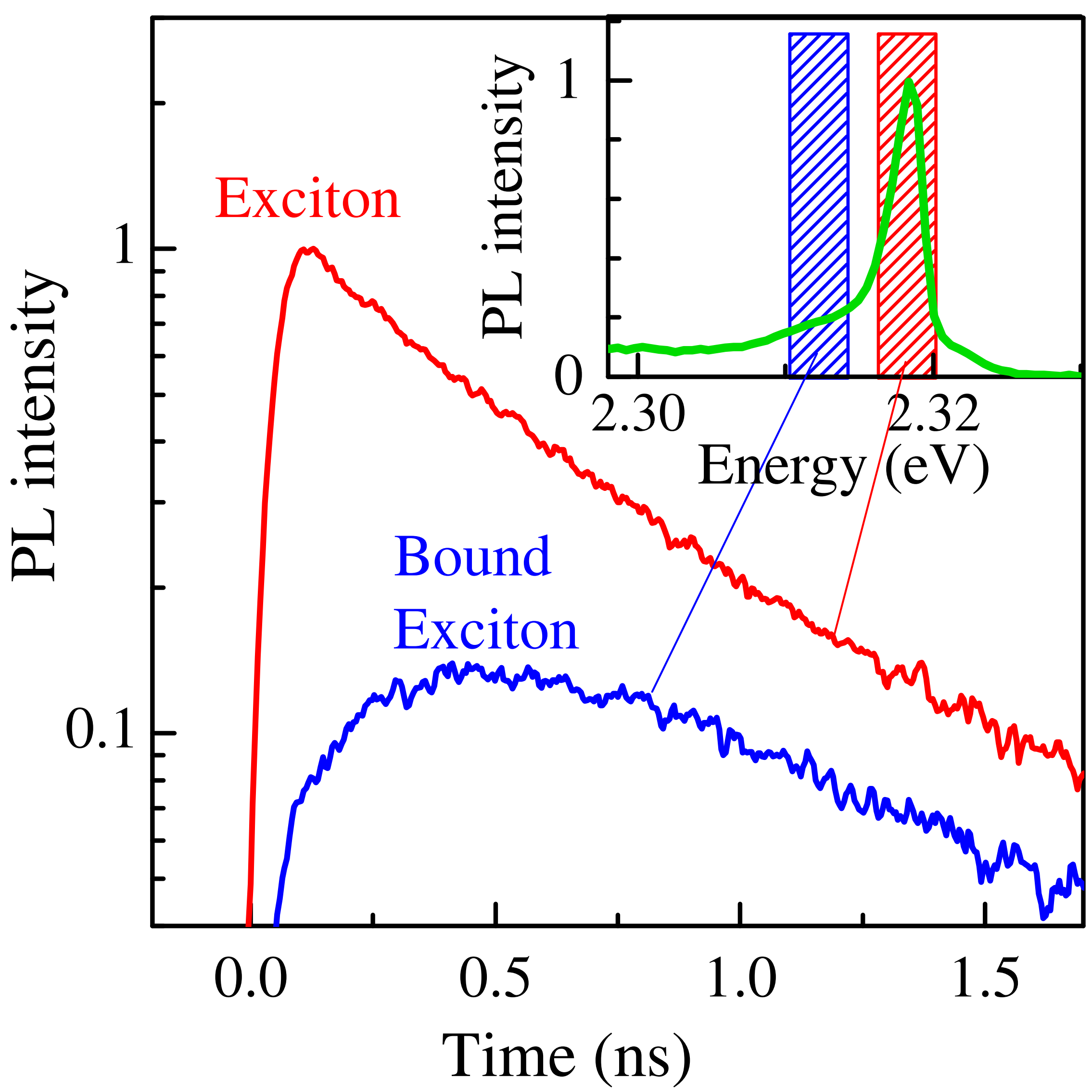

Supplementary Figure 2: Photoluminescence dynamics for the spectral regions shaded in the inset and corresponding to the excitons (red line) and bound excitons (blue line). The excitation photon energy is 3.263 eV, $T = 10$ K. Source data are provided as a Source Data file.

Isotope	Spin	μ_I/μ_N	Abundance β	A_e (μeV)	A_h (μeV)
$^{133}_{55}$Cs	$7/2$	2.58	100%	$-^a$	$-^a$
$^{206}_{82}$Pb	0	0	24.1%	$-^b$	$-^b$
$^{207}_{82}$Pb	$1/2$	0.58	22.1%	$-^a$	100 (20)
$^{208}_{82}$Pb	0	0	52.4%	$-^b$	$-^b$
$^{79}_{35}$Br	$3/2$	2.1	50.7%	7 (3.5)	$-^a$
$^{81}_{35}$Br	$3/2$	2.27	49.3%	7 (3.5)	$-^a$

[a]The contribution of its orbitals to the Bloch function in negligible.

[b]This isotope has spin 0 and the hyperfine interaction is absent.

Supplementary Table 1: Parameters of the hyperfine interaction in $CsPbBr_3$. The values of A_e and A_h are given following the estimates in the text, the values in parenthesis are normalized to the abundance. Only isotopes with significant abundance are included.

Supplementary Note 1. BAND STRUCTURE AND ORBITAL COMPOSITION OF BLOCH FUNCTIONS

Bulk halide perovskite semiconductors are described by the chemical formula ABX_3. Here A is the organic or inorganic cation (center of the unit cell), B is the metal atom (e.g., Pb) and X is the halogen atom (e.g., Br). To be specific, we consider $CsPbBr_3$ and, for simplicity, its cubic modification described by the point group O_h. Note that the crystal becomes orthorhombic at low temperatures, but here we ignore the minor modifications of the selection rules and of the hyperfine interaction for different phases of the crystal. The direct band gap is formed at the R point of the Brillouin zone (the corner of the cube), the symmetry of the R valley is the same as that of the Γ point, i.e., O_h.

The orbital Bloch functions of the *valence band top* are invariant (R_1^+ representation) and of the *conduction band bottom* are three functions transforming according to the R_4^- representation (as vector components)[1,2]. With account for the spin and spin-orbit coupling (R_6^+ is the spinor representation for spin-$1/2$ states) one obtains

$$\text{valence band}: \quad R_1^+ \times R_6^+ = R_6^+, \tag{1}$$

$$\text{conduction band}: \quad R_4^- \times R_6^+ = R_6^- + R_8^-. \tag{2}$$

The bottom of the conduction band has R_6^- symmetry, see Refs.[1,3].

According to Ref.[1], the valence band is mainly composed by the s-orbitals of the metal, $|\mathcal{S}_0\rangle$, with admixture of the halogen p-orbitals (a combination $\propto |\mathcal{X}_1\rangle + |\mathcal{Y}_2\rangle + |\mathcal{Z}_3\rangle$ with appropriate phase choice). For the conduction band the main contribution comes from the p-orbitals of the metal, $|\mathcal{X}_0\rangle$, $|\mathcal{Y}_0\rangle$, $|\mathcal{Z}_0\rangle$ with a slight ($\lesssim 1\%$) admixture of the s-orbitals of the halogen $|\mathcal{S}_1\rangle$.

Supplementary Note 2. SPIN POLARIZATION OF RESIDENT CHARGE CARRIERS BY CIRCULARLY POLARIZED LIGHT

The resonant absorption of circularly polarized light induces transitions from the R_6^+ valence band to the R_6^- conduction band. Both bands are two-fold degenerate in the spin and electron-

hole pairs with spin orientations $(+1/2_\mathrm{e}, +1/2_\mathrm{h})$ or $(-1/2_\mathrm{e}, -1/2_\mathrm{h})$ are generated, respectively, by the σ^+ and σ^- polarized photons. The subscripts e and h denote electrons and holes and we use the hole representation here (the spin of the unoccupied valence band state is opposite to the hole spin).

The photocreated electron-hole pairs or excitons can transfer their spin polarization to the resident charge carriers. The polarization mechanisms were studied in detail for III-V and II-VI semiconductors, see Refs. 4,5 for review. The mechanisms are related to either the formation of bound three particle complexes (positively or negatively charged excitons, also termed as trions) or to the exchange scattering of the excitons by the resident carriers. Particularly, in the latter case the scattering of the spin-polarized exciton with an unpolarized electron and hole results in the exchange of the identical carriers and in the transfer of the spin polarization to the resident electrons or holes. After exciton recombination the spin polarization remains in the system of resident carriers.

Supplementary Note 3. HYPERFINE INTERACTION IN PEROVSKITES

For localized carriers the spin-orbit coupling effects are suppressed due to the quenching of the orbital motion. Indeed, the odd in electron wavevectors contributions to the spin-orbit Hamiltonian vanish as a result of averaging over the localized electron wavefunctions[5,6]. Thus, the hyperfine coupling of electron or hole spins with host lattice nuclei are the prime candidates for driving the spin dynamics[6]. The nuclear spins experience the Knight field from the spin polarized charge carriers and, in turn, the charge carriers experience the Overhauser field induced by the nuclei.

The hyperfine interaction between the charge carriers and nuclei provides a transfer of spin angular momentum from the electrons or holes to the nuclei and thereby results in a dynamical nuclear polarization. Generally, the hyperfine coupling Hamiltonian can be written in the phenomenological form of a scalar product of the nuclear and charge carrier spins (see below for details):

$$\mathcal{H}_\mathrm{hf} = A_\mathrm{e(h)} v_0 |\varphi_\mathrm{e(h)}(\boldsymbol{R})|^2 (\mathbf{I} \cdot \mathbf{S}_\mathrm{e(h)}), \tag{3}$$

where $\mathbf{I}$ is the nuclear spin, $\mathbf{S}_{\rm e}$ ($\mathbf{S}_{\rm h}$) is the electron (hole) spin, $A_{\rm e}$ ($A_{\rm h}$) is the corresponding interaction constant. The factors v_0, the unit cell volume, and $|\varphi_{\rm e(h)}(\boldsymbol{R})|^2$, the absolute square of the envelope function of the charge carrier at the nucleus position, are introduced in Eq. (3) to make $A_{\rm e(h)}$ dimensionless. The anisotropic terms allowed in cubic semiconductors for $I > 1/2$ are usually small and disregarded hereafter.

In the presence of an external magnetic field, $\mathbf{B}$, the hyperfine interaction [Eq. (3)] provides the spin transfer between electron (hole) and nuclear spins. In the experimental configuration shown in Fig. 3(b) in the main text there is a non-zero component of the charge carrier spin $\mathbf{S}_{\rm e(h)}$ onto the magnetic field $\mathbf{B}$. Flip-flop hyperfine processes give rise to the dynamical nuclear spin polarization $\langle \mathbf{I} \rangle$ in the form[6]

$$\langle \mathbf{I} \rangle = \ell_{\rm e(h)} \frac{4I(I+1)}{3} \frac{\mathbf{B}(\mathbf{B}\cdot\mathbf{S}_{\rm e(h)})}{B^2}, \tag{4}$$

where $\ell_{\rm e(h)} \leq 1$ is the leakage factor characterizing the losses of nuclear spin polarization due to relaxation processes other than the hyperfine coupling.

Via the hyperfine interaction, the polarized nuclear spins produce the Overhauser field

$$\mathbf{B}_{\rm N,e(h)} = (g_{\rm e(h)}\mu_{\rm B})^{-1} \sum_j A^j_{\rm e(h)} v_0 |\varphi_{\rm e(h)}(\boldsymbol{R}_j)|^2 \langle \mathbf{I}_j \rangle, \tag{5}$$

where the summation is carried out over all nuclei, so that the index j includes all chemical elements, all isotopes of the element abundant in the sample, as well as all positions $\boldsymbol{R}_j$ of the nuclei. Under the standard assumption of a uniform nuclear spin polarization $\langle \mathbf{I} \rangle$ Eq. (5) can be written in a simple form as

$$\mathbf{B}_{\rm N,e(h)} = (g_{\rm e(h)}\mu_B)^{-1} \sum_i A^i_{\rm e(h)} \langle \mathbf{I}_i \rangle, \tag{6}$$

where the sum is carried out over the different elements and isotopes denoted by the subscript i. Indeed, the summation over unit cells assuming homogeneous nuclear polarization, can be transformed to an integral as

$$\sum_j v_0 |\varphi_{\rm e(h)}(\boldsymbol{R}_j)|^2 = N_{\rm iso} \int d\boldsymbol{R}_j |\varphi_{\rm e(h)}(\boldsymbol{R}_j)|^2 = 1, \tag{7}$$

where $N_{\rm iso}$ is the number of corresponding isotopes in the unit cell. In line with the smooth envelope method, we assume that $\varphi_{\rm e(h)}(\boldsymbol{R}_j)$ does not vary much on the scale of the lattice constant.

The Overhauser field $\mathbf{B}_{\rm N}$ adds up to the external field changing the frequency of the carrier spin precession. The direction of $\mathbf{B}_{\rm N,e(h)}$ is determined by the sign of the hyperfine coupling constant $A_{\rm e(h)}$ and the direction of $\mathbf{I}$ which, in turn, is governed by $\mathbf{S}_{\rm e(h)}$, i.e., it can be adjusted by varying the light helicity. We performed measurements for both σ^+ and σ^- circularly polarized pumping, inducing the opposite directions of $\mathbf{B}_{\rm N,e(h)}$. Measuring the change of the electron and hole spin precession frequencies we detected the nuclear field.

Figure 3b in the main text shows that the spin precessions for opposite pump polarizations acquire a small, but still reliably detectable phase shift which increases with time delay (inset to Fig. 3b in the main text). This means that the precession frequencies for σ^+ and σ^- pumping are different. An accurate fit to the experimental data shows that the nuclear field acting on the electron spins is $|\mathbf{B}_{\rm N,e}| = 1.0 \pm 0.8$ mT and that on the hole spins is $|\mathbf{B}_{\rm N,h}| = 3.1 \pm 0.5$ mT. We conclude that the hyperfine interaction mostly affects the valence band hole spins.

This experimental result may seem surprising compared with the widely studied III-V and II-VI semiconductors where the hyperfine coupling is dominated by the conduction band electrons[7–10]. Our theoretical analysis, however, confirms that in perovskite like $CsPbBr_3$ the hyperfine coupling for the valence band holes is stronger compared to the conduction band electrons. To that end we consider in more detail the atomic orbital composition of the Bloch states for electrons and holes. The valence band states in the vicinity of the R-point of the Brillouin zone, where the direct band gap is formed, is mainly composed by s-type atomic orbitals of the metal (lead in our case) with an admixture of p-type atomic orbitals of the halogen (i.e., bromine), see, e.g., Ref.[1] and Supplementary Note Supplementary Note 1 for details. The leading contribution to the hyperfine coupling is provided by the Fermi contact interaction for the s-type orbitals, which does not vanish at the positions of the nuclei. By contrast, the conduction band is mainly formed from the p-type metal orbitals with a slight admixture of the s-type orbitals of the halogen. The magnetic dipole-dipole interaction for p-type orbitals is about an order of

magnitude weaker[8,10].

The hyperfine interaction constants for $CsPbBr_3$ can be estimated as follows. In the **valence band** the hyperfine coupling is dominated by the contact interaction with the the lead atoms. The constant $A_{\rm h}^{(0)}$ calculated per isotope, i.e., disregarding the abundance, can be written as[9]:

$$A_{\rm h}^{(0)} = \frac{16\pi\mu_{\rm B}\mu_I}{3I} v_0^{-1}|\mathcal{S}_0(0)|^2, \quad (8)$$

where $\mathcal{S}_0(\boldsymbol{r})$ is the Bloch function at the nucleus position normalized per volume of the unit cell, $\int_{v_0} |\mathcal{S}_0(\boldsymbol{r})|^2 d\boldsymbol{r} = v_0$, $\mu_{\rm B}$ is the Bohr magneton, μ_I is the nuclear magnetic moment, I is the spin of the nucleus. It is important to note that the transformation from the electron to the hole representation results in the inversion of both the direction of spin and the energy axis, leaving the hyperfine constant sign the same. That is why we can use the electron representation for evaluation of the hyperfine coupling for holes.

For holes the relevant isotope is ^{207}Pb with an abundance of about 22%, the nuclear spin $I = 1/2$ and $\mu_I = 0.58\mu_{\rm N}$, where $\mu_{\rm N} \approx 7.62$ MHz/T is the nuclear magneton. The hyperfine interaction can be estimated from the atomic constants[11,12]. From Ref.[11] (which uses an approach that typically overestimates the value, as known from the comparison for III-V semiconductors) we have (per nucleus):

$$A_{\rm h}^{(0)} = 107\ \mu\text{eV}. \quad (9)$$

Note that inclusion of the so-called Mackey-Wood correction gives a ~ 3.15-fold enhancement up to 336 μeV. From Ref.[12] (which approach typically underestimates the hyperfine coupling), disregarding the anisotropy factor we have :

$$A_{\rm h}^{(0)} = 78\ \mu\text{eV}. \quad (10)$$

Thus, we take $A_{\rm h}^{(0)} \approx 100$ μeV as a conservative estimate which is also in agreement with estimates and measurements of the Fermi contact interaction in $Pb_{1-x}Sn_xTe$[13]. Taking into account that the natural abundance of ^{207}Pb is $\beta \approx 22\%$[14], we finally have $A_{\rm h} \approx 20$ μeV.

Note that the dipole-dipole interaction with ^{79}Br and ^{81}Br (both $I = 3/2$) can be estimated as $A_{\rm h}^1 \sim |C_p|^2 \times 7$ μeV, where C_p ($|C_p| \ll 1$) is the admixture of the p-shell of Br to the s-shell of Pb in the valence band Bloch function, therefore, it can be disregarded.

For the **conduction band** states it is sufficient to account for the dipole-dipole interaction with the bromine nuclei only. Estimates based on Refs.[11,12] give $A_\mathrm{e} \approx 7\ \mu$eV. We use this constant for both the $^{79}_{35}$Br and $^{81}_{35}$Br isotopes whose spin is the identical and whose total abundance is 100%. The parameters of the hyperfine interaction are summarized in Supplementary Table 1.

For complete nuclear polarization, where $|\langle \mathbf{I} \rangle| = I$ according to our estimates after Eq. (6) the maximum Overhauser fields read

$$|B_{\mathrm{N,h}}^{\mathrm{max}}| \approx 230\ \mathrm{mT}, \quad |B_{\mathrm{N,e}}^{\mathrm{max}}| \approx 280\ \mathrm{mT} \tag{11}$$

for the holes and electrons, respectively. Interestingly, the maximal Overhauser field is slightly larger for the conduction band despite the smaller hyperfine coupling constants. This is because there are three Br nuclei in the unit cell with $I = 3/2$ and because of the smaller abundance of Pb. However, the dynamical nuclear polarization process is controlled by individual spin-flips, where the hyperfine interaction with holes prevails. The flip-flop of Br nuclei with the electron spin is controlled by the much smaller individual interaction constant. Thus, we expect the dynamical nuclear polarization of Br to be weaker, in agreement with the smaller leakage factors obtained in the experiment: $\ell_\mathrm{h} \sim 0.08$ for the valence band and $\ell_\mathrm{e} \sim 0.02$ for the conduction band. We took the leakage factors from the measured Overhauser field using the estimates in Eqs. (4) and (11) and considering the angle of $\sim 75^\circ$ between the magnetic field and the light propagation axis.

Supplementary Note 4. ZEEMAN HAMILTONIAN AND g-FACTOR OF CARRIERS AND EXCITONS

We define the effective exciton Zeeman Hamiltonian in the form[15–18]

$$\widehat{H}_Z = \mu_\mathrm{B} g_\mathrm{e} \left(\boldsymbol{S}_\mathrm{e} \boldsymbol{B} \right) + \mu_\mathrm{B} g_\mathrm{h} \left(\tilde{\boldsymbol{S}}_\mathrm{h} \boldsymbol{B} \right). \tag{12}$$

Here μ_B is the Bohr magneton, g_e and g_h are the electron and hole g-factors, respectively, $S_\mathrm{e} = \tilde{S}_\mathrm{h} = \pm 1/2$ are the electron and hole spins. Note that we use the standard representation for the exciton Hamiltonian where the electron part is taken in the electron and the hole part is

taken in the hole presentation, that is, why the Hamiltonian is the sum of the electron and the hole contributions. In this definition the g-factors are positive both for electrons and holes if the ground state is the state with spin projection $s_z = -1/2$ onto the direction of the magnetic field. Note that just like for the hyperfine coupling, the value and the sign of the hole g-factor is the same, both for the electron and hole representations. The exciton g-factor is defined as

$$g_{\mathrm{X}} = \frac{E_{\sigma^+} - E_{\sigma^-}}{\mu_{\mathrm{B}} B}. \tag{13}$$

In case of the perovskites the exciton g-factor is the sum of the electron and hole g-factors: $g_{\mathrm{X}} = g_{\mathrm{e}} + g_{\mathrm{h}}$. The slight discrepancy between the values of the g_{X} inferred directly from optical spectroscopy, on the one hand, and from the sum of g_{e} and g_{h}, on the other hand, may be attributed to the effects of the electron-hole exchange interaction and band non-parabolicity which gives rise to a renormalization of the g-factors of the charge carriers in the exciton[19].

Supplementary Note 5. ANALYSIS OF THE SPIN INERTIA DATA AND POLARIZATION RECOVERY CURVES

In order to analyze the magnetic field dependence of the polarization recovery curves we employ the model developed in Ref. 20. It accounts for both the spin precession in the field of the nuclear fluctuations and the finite correlation time of the hole at a localization site, τ_{c}. We consider the case of small modulation frequencies where the spin inertia is not important. Introducing ω_{N} as the characteristic fluctuation of the Overhauser field we obtain

$$PRC \propto \left(\frac{1}{\tau_{\mathrm{s}}} + \frac{\omega_{\mathrm{N}}^2 \tau_{\mathrm{c}}}{1 + (\omega_{\mathrm{L,h}} \tau_{\mathrm{c}})^2} \right)^{-1}. \tag{14}$$

We recall that $\omega_{\mathrm{L,h}}$ is the hole spin precession frequency in the external magnetic field and τ_{s} is the spin relaxation time unrelated to the hyperfine coupling. Equation (14) holds provided $\omega_{\mathrm{N}} \tau_{\mathrm{c}} \ll 1$ or $\omega_{\mathrm{L,h}} \gg \omega_{\mathrm{N}}$. The fit after Eq. (14) with τ_{c} and ω_{N} being free parameters and $\tau_{\mathrm{s}} = 50$ ns (inferred from the spin inertia measurements) gives the correlation time $\tau_{\mathrm{c}} \approx 2.1$ ns and the nuclear field fluctuation $\delta B_{\mathrm{N}} = \hbar \omega_{\mathrm{N}} / (g_{\mathrm{h}} \mu_{\mathrm{B}}) \approx 6.6$ mT. In this case $\omega_{\mathrm{N}} \tau_{\mathrm{c}} \approx 0.9$ and Eq. (14) is not applicable at $B \lesssim \delta B_{\mathrm{N}}$. Thus, to confirm the results, we have performed the full

calculation after the model of Ref. 20 shown by the red curve in Supplementary Figure 1 for the same parameter values. Very good agreement between the calculations and experimental data is observed.

The value of the nuclear spin fluctuation $\delta B_{\mathrm{N}} \approx 6.6$ mT allows us to make a crude estimate of the hole localization length. To that end we evaluate ω_{N}, assuming independent contributions of the Pb isotopes as

$$\omega_{\mathrm{N}} = \sqrt{\frac{2}{3\hbar^2} I(I+1)} \frac{A_{\mathrm{h}}^{(0)} \sqrt{N_{\mathrm{Pb}}}}{N_{\text{cells}}}, \tag{15}$$

where N_{cells} is the number of cells within the hole localization volume, $N_{\mathrm{Pb}} = \beta N_{\text{cells}}$ is the number of lead isotopes with non-zero spin within the hole localization volume. It follows from Eq. (15) that for the experimental value of $\omega_{\mathrm{N}} = g_{\mathrm{h}} \mu_{\mathrm{B}} \delta B_{\mathrm{N}}/\hbar \approx 0.44$ ns^{-1} the localization volume contains $N_{\text{cells}} \approx 8 \times 10^3$ unit cells, which gives the localization length $L_{\text{loc}} = a_0 N_{\text{cells}}^{1/3} \approx 11.5$ nm.

Supplementary Note 6. PHOTOLUMINESCENCE DYNAMICS OF FREE AND BOUND EXCITONS

We also measure the photoluminescence (PL) dynamics of the low-energy shoulder in the PL spectrum corresponding to the bound exciton. It is shown by the blue line in Supplementary Figure 2 in comparison to the PL dynamics of the exciton (the red line). Bound exciton PL shows increase with ~ 0.4 ns time which can be attributed to capture of the excitons by impurities. The decay time of the bound exciton PL (~ 0.9 ns) is the same as the exciton lifetime (~ 0.9 ns) and is much shorter than maximal spin dephasing time T_2^* of electrons (~ 5.2 ns) and longitudinal spin relaxation time T_1 (~ 50 ns) indicating that the observed spin dynamics is relater neither to excitons nor to bound excitons.

Supplementary References

---

[1] Boyer-Richard, S., Katan, C., Traore, B., Scholz, R., Jancu, J.-M., & Even, J. Symmetry-based tight binding modeling of halide perovskite semiconductors. *J. Phys. Chem. Lett.* **7**, 3833-3840 (2016).

[2] Even, J. Pedestrian guide to symmetry properties of the reference cubic structure of 3D all-inorganic and hybrid perovskites. *J. Phys. Chem. Lett.* **6**, 2238-2242 (2015).

[3] Becker, M. A. *et al.* Bright triplet excitons in caesium lead halide perovskites. *Nature* **553**, 189-193 (2018).

[4] Yakovlev, D. R. & Bayer, M. in *Spin physics in semiconductors*, ed. M. I. Dyakonov (Springer International Publishing AG, 2017) pp. 155-206.

[5] Glazov, M. M. Coherent spin dynamics of electrons and excitons in nanostructures (a review). *Phys. Solid State* **54**, 1-27 (2012).

[6] Meier, F. & Zakharchenya, B. P. eds., *Optical orientation* (Horth-Holland, Amsterdam, 1984).

[7] Paget, D., Lampel, G., Sapoval, B., & Safarov, V. I. Low field electron-nuclear spin coupling in gallium arsenide under optical pumping conditions. *Phys. Rev. B* **15**, 5780-5796 (1977).

[8] Gryncharova, E. I. & Perel', V. I. Relaxation of nuclear spins interacting with holes in semiconductors. *Sov. Phys. Semicond.* **11**, 997 (1977).

[9] Chekhovich, E. A. *et al.* Element-sensitive measurement of the hole-nuclear spin interaction in quantum dots. *Nat. Phys.* **9**, 74-78 (2013).

[10] Chekhovich, E. A., Ulhaq, A., Zallo, E., Ding, F., Schmidt, O. G. & Skolnick, M. S. Measurement of the spin temperature of optically cooled nuclei and GaAs hyperfine constants in GaAs/AlGaAs quantum dots. *Nat. Mater.* **16**, 982-986 (2017).

[11] Morton, J., & Preston, K. Atomic parameters for paramagnetic resonance data. *J. Magn. Res.(1969)* **30**, 577-582 (1978).

[12] Koh A. & Miller D. Hyperfine coupling constants and atomic parameters for electron paramagnetic resonance data. *Atomic Data and Nuclear Data Tables* **33**, 235-253 (1985).

[13] Hewes, C. R., Adler, M. S., & Senturia, S. D. Nuclear-magnetic-resonance studies in PbTe and $Pb_{1-x}Sn_xTe$: An experimental determination of kp band parameters and magnetic hyperfine constants. *Phys. Rev. B* **7**, 5195-5212 (1973).

[14] Fuller, G. H. Nuclear Spins and Moments. *J. Phys. Chem. Ref. Data* **5**, 835-1092 (1976).

[15] Kiselev, A. A. & Moiseev, L. V. Zeeman splitting of heavy-hole states in III-V and II-VI heterostructures. *Phys. Solid State* **38**, 866 (1996).

[16] Durnev, M. V., Glazov, M. M., & Ivchenko, E. L. Giant Zeeman splitting of light holes in GaAs/AlGaAs quantum wells. *Physica E* **44**, 797-802 (2012).

[17] Yu, Z. G. Effective-mass model and magneto-optical properties in hybrid perovskites. *Sci. Rep.* **6**, 28576 (2016).

[18] Ramade, J. *et al.* Fine structure of excitons and electron-hole exchange energy in polymorphic $CsPbBr_3$ single nanocrystals. *Nanoscale* **10**, 6393-6401 (2018).

[19] P. S. Grigoryev *et al.* Inversion of Zeeman splitting of exciton states in InGaAs quantum wells *Phys. Rev. B* **93**, 205425 (2016).

[20] D. S. Smirnov *et al.* Theory of spin inertia in singly-charged quantum dots. *Phys. Rev. B* **98**, 125306 (2018).
